# Microbiological Profiling of Menstrual Blood Aspirated from the Uterus in Patients Undergoing Frozen Embryo Transfer

**DOI:** 10.3390/diagnostics16091403

**Published:** 2026-05-06

**Authors:** Mark Jain, Elena Mladova, Pavel Zalepaev, Margarita Gundobina, Alexander Klimov, Liya Shcherbakova, Larisa Samokhodskaya, Olga Panina

**Affiliations:** 1 University Clinic, Lomonosov Moscow State University, 119992 Moscow, Russia; zalepaeff@gmail.com (P.Z.); liya.fbm@yandex.ru (L.S.); slm61@mail.ru (L.S.); olgapanina@yandex.ru (O.P.); 2 Institute of Reproductive Medicine “REMEDI”, 123100 Moscow, Russia; e.mladova@remediclinic.ru; 3 Faculty of Medicine, Lomonosov Moscow State University, 119992 Moscow, Russia; gundobinamargarita@gmail.com (M.G.); aleksander.klimov1@gmail.com (A.K.)

**Keywords:** endometrial receptivity, microbiota, menstrual blood, embryo transfer, in vitro fertilization, assisted reproduction

## Abstract

**Background**: There is growing evidence that uterine microbiota might be linked to endometrial receptivity (ER) and affect the outcome of assisted reproductive technology (ART) procedures. Owing to the invasive nature of endometrial sampling, the evaluation of microbiota in this biomaterial is only possible outside the embryo transfer (ET) cycle. However, menstrual blood might be the key to overcoming this challenge as it can be safely aspirated from the uterine cavity at the beginning of the target ET cycle. This study aimed to evaluate the association of the microbiological profiles of menstrual blood with ER in patients undergoing frozen ET. **Methods:** Menstrual blood was obtained from 98 individuals scheduled for frozen ET in a private ART clinic (ET success rate–50%). DNA was isolated from menstrual sediment and analyzed using a multiplex quantitative PCR assay designed to identify 28 relevant microbial taxa and 3 Herpesviridae viruses. **Results:** Bacterial DNA was detected in 75.5% of samples. There were no associations between the abundance of individual microbial taxa and the outcome of ET, and the same was true for Shannon’s α-diversity indices (*p* > 0.05). However, *Candida* spp. and Enterobacteriaceae were detected exclusively in patients with negative ET outcomes (*p* = 0.028). Individuals with recurrent implantation failure had a significantly lower abundance of *Lactobacillus* spp. than the rest (0.0 [0.0; 7.4] vs. 2.8 [0.0; 91.9] %, *p* = 0.024). **Conclusions:** Menstrual blood aspirated directly from the uterus is a promising biomaterial for endometrial microbiological profiling. Upon further investigation, its analysis might become a useful tool in managing infertile patients scheduled for ART treatment.

## 1. Introduction

The human microbiota, often referred to as “the additional organ”, provides approximately 150 times more genetic information than our entire genome [1]. It is estimated that the microbiota makes up to 0.3% of a human’s total mass, and there is one microorganism for every cell in the human body [2]. As a biological species, *Homo sapiens* evolved in the context of constant crosstalk between our non-sterile organs and various microorganisms. A proper interaction between microbiota and human tissue is essential for the metabolism of nutrients and drugs, regulation of the immune system, and protection against pathological agents [3]. There is emerging evidence that microbiota might be linked to many other aspects of our health, including the development of psychiatric conditions, tumor growth, and reproductive system functioning [4,5,6].

Infertility is defined as the failure to achieve pregnancy after engaging in regular unprotected sexual intercourse for at least a year [7]. This condition affects over 180 million people worldwide, and it is estimated that approximately 17% of couples of reproductive age will encounter infertility at some point in their lives, which largely explains the growth of the assisted reproductive technologies (ART) market [8]. Female factors are the main ones in approximately 50% of infertility cases, and impaired endometrial receptivity (ER) is often referred to as the most impactful cause of this condition [9]. ER is an essential feature of the endometrium that is responsible for a complex process that provides the embryo with appropriate conditions to attach, invade, and develop [10].

For many years, it was believed that the endometrium is sterile due to the cervical barrier; thus, microbiota was often overlooked in the context of ER studies [11,12]. However, recent data suggest that not only is the endometrium not sterile, but its microbiota may also be an important factor influencing ER [13]. Unfortunately, several methodological challenges prevent the translation of these findings into clinical practice and cause certain discrepancies. First, sampling of endometrial tissue using various forms of biopsy (including the aspiration of endometrial secretions) is considered quite invasive; thus, it is not allowed within the embryo transfer (ET) cycle. Therefore, researchers have to rely on data obtained in menstrual cycles preceding the one in which IVF will be performed. At the same time, there is insufficient evidence that microbiological profiles are stable from cycle to cycle. Second, it is difficult to rule out contamination by microbiota from the lower parts of the reproductive tract, given the upward nature of endometrial sampling. Third, modern high-sensitivity microbiome analysis techniques are prone to the “splashome” effect (contamination of neighboring wells on the plate by microbial DNA from samples with a high abundance of these molecules) [14].

We hypothesize that menstrual blood might become a feasible alternative to invasively collected endometrial tissue for microbiological analysis. This biomaterial remains in a state of prolonged contact with the uterus, which may result in it containing genetic material of microorganisms populating this environment. Menstrual blood might be painlessly aspirated directly from the uterus (the cervix is dilated and passable during menstruation), largely preventing contamination by vaginal and cervical microbiota. Moreover, this biomaterial could be collected and rapidly analyzed at the beginning of the respective ET cycle, potentially providing opportunities to reschedule the ART procedure based on the data obtained. Our recent pilot study has proved that sampling of menstrual blood in the ET cycle is safe and does not negatively impact the ET success rate in infertile patients; this biomaterial contains enough microbial DNA for the corresponding analysis (even with a conservative detection call threshold); its microbiological profiles might be linked to several infertility factors [15].

Therefore, this study aimed to evaluate the association of microbiological profiles of menstrual blood aspirated directly from the uterus with ER in patients undergoing frozen ET.

## 2. Materials and Methods

### 2.1. General Information

This prospective study included 98 patients scheduled for frozen ET in a private ART clinic. Patient enrollment was conducted from January 2021 to October 2025. We did not apply any other inclusion/exclusion criteria; thus, our cohort resembles the general population that receives treatment in an ART clinic. Frozen ET and other ART procedures were performed in strict accordance with national guidelines for ART. Only high-quality blastocysts were selected for ET (Gardner score of ≥2 BB). Study participants were divided into two groups based on the outcome of frozen ET (pregnant and nonpregnant). Clinical pregnancy was verified using human chorionic gonadotropin testing with subsequent ultrasonographic assessment (visualization of ≥1 gestational sac). Pregnancy outcome was not evaluated, as this variable is dependent on various factors that do not fit the scope of this work. The relevant clinical and demographic features of the study participants are presented in Table 1. Table S1 presents detailed and depersonalized data for each individual.

### 2.2. Sampling, Processing, and Analysis

A detailed description of the methodology used for the microbiological profiling of menstrual blood is available in our previously published pilot study [15]. In brief, menstrual blood was aspirated directly from the uterine cavity on days 2–3 of the frozen ET cycle using an ET catheter. DNA was extracted from menstrual sediment resuspended in a sterile 0.9% saline solution using the QIAamp DNA Mini Kit (Qiagen GmbH, Hilden, Germany). The microbiological profiling included analysis of 28 microbial and 3 viral taxa common for the female reproductive tract using validated multiplex assays “Femoflor 16”, “TNC Complex”, and “Herpes Multiplex” on a DT-Prime real-time PCR instrument (DNA-Technology, Moscow, Russia). These assays included primers and probes for *Homo sapiens* DNA to control the quality of biomaterial collection and processing (in accordance with the manufacturer’s instructions, the threshold was set to 10^3^ copies per reaction mixture). The influence of the so-called “kitome” (contamination of the final analytical mixtures by bacteria present on the reagents and consumables utilized at various stages of the sample handling process) was assessed by exposure of negative control sterile 0.9% saline solutions to the above-mentioned procedures at all stages of the microbiological profiling. To diminish the influence of any possible “splashome” and contamination of samples by microbial and viral DNA from the lower parts of the reproductive tract, a conservative threshold of 10^3^ copies of analyzed target per reaction mixture was applied for the detection calls during real-time PCR analysis. According to the information provided by the manufacturer of the assays, this threshold is characterized by a sensitivity of 97% and a specificity of 97%. Total bacterial load was quantified based on the amplification of a conserved region of bacterial DNA. The results of microbiological profiling for each taxon analyzed are presented as “abundance” (% of total bacterial load). Due to the limitations of the multiplex real-time PCR assays used, the data are presented at the family level for some microorganisms and at the genus level for others.

### 2.3. Statistical Analysis

Data were processed using IBM SPSS Statistics 27.0 (IBM Corp., Armonk, NY, USA). Distribution normality was assessed using the Shapiro–Wilk test. As the normal distribution was absent for all tested variables, nonparametric statistical tests were applied. Continuous variables are presented as median [quartile 1; quartile 3] unless stated otherwise. Categorical variables were compared using Fisher’s exact test, whereas continuous variables were compared using Mann–Whitney’s U test. Relationships between continuous variables were assessed using Spearman’s rank correlation coefficient (r_S_), which was interpreted according to Chaddock’s scale. Logistic regression was used to evaluate the role of confounding factors. Shannon’s index was calculated to describe the α-diversity of the analyzed communities. As the quantity of microbial taxa included in the microbiological profiling in this study was limited, Shannon’s indices only reflect the α-diversity within the boundaries of the applied multiplex real-time PCR assays; thus, they should not be directly compared to data obtained in 16S rRNA sequencing studies. *p*-values below 0.05 were considered statistically significant. Because of the exploratory design of the study, strict correction for multiple comparisons (e.g., Bonferroni correction) was not applied. Instead, we assessed the false discovery rate (FDR) using the Benjamini–Hochberg procedure at an FDR of 0.2. *p*-values below 0.05 that did not pass the threshold for this correction for multiple comparisons are marked with an asterisk “*”.

## 3. Results

### 3.1. Sample Quality

All samples included in the analysis met the quality control criteria and contained human DNA above the previously mentioned threshold of 10^3^ copies per reaction mixture (6.3 × 10^5^ [3.2 × 10^5^; 1.5 × 10^6^] copies per reaction mixture). At the same time, none of the negative controls used to verify the absence of the “kitome” effect exhibited the presence of microbial and viral DNA. Total bacterial load analysis revealed that bacterial DNA above the detection call threshold (10^3^ copies per reaction mixture) was present only in 75.5% of menstrual blood DNA samples.

Among the positive samples, the total bacterial load varied significantly (5.9 × 10^3^ [2.5 × 10^3^; 2.7 × 10^4^] copies of bacterial genomes per reaction mixture). No correlation was found between the level of human DNA and total bacterial load (r_S_ = 0.195; *p* > 0.05), highlighting that the absence of bacterial DNA could not be explained by the poor efficiency of DNA extraction.

### 3.2. Association with Frozen ET Outcomes

**Figure 1:**
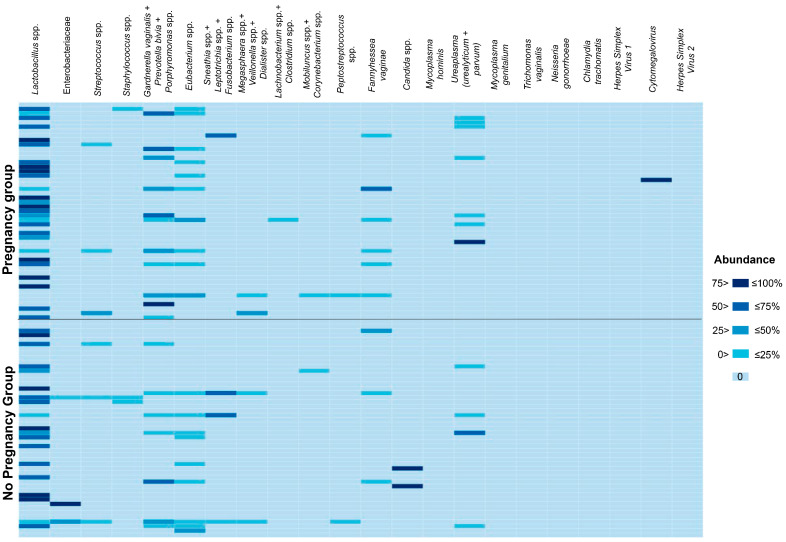
Heatmap summarizing microbiological profiling results for menstrual blood. The vertical axis corresponds to samples from study participants, whereas the horizontal axis corresponds to the data for each taxon analyzed. Data are presented as abundance (DNA concentration for each analyte normalized by total bacterial load, %). The scale of the heatmap was segmented to facilitate the interpretation of results in cases with low abundances. The intensity of cell coloration corresponds to the abundance value according to the scale of the heatmap. For convenience, the levels of *Candida* spp. DNA was added to the total bacterial load values. Some closely related taxa were analyzed collectively due to the peculiarities of the applied real-time PCR assay. In such cases, a “+” sign joins them.

The results of the microbiological profiling of menstrual blood are summarized as a heatmap in Figure 1. Abundances of analyzed taxa for each sample included in the study are shown in Table S1. Both qualitative and quantitative comparisons of microbiological profiling results demonstrated the absence of any significant differences between patients who achieved and did not achieve pregnancy after frozen ET for any of the analyzed taxa (*p* > 0.05). The same was true when the cohort was stratified based on the prolongation of pregnancy up to 12 weeks (*p* > 0.05). It is worth noting that, given the low detection rates of most taxa, achieving sufficient statistical power for these comparisons was possible only for *Lactobacillus* spp., *Eubacterium* spp., *Gardnerella vaginalis*, *Prevotella bivia*, and *Porphyromonas* spp.

**Figure 2:**
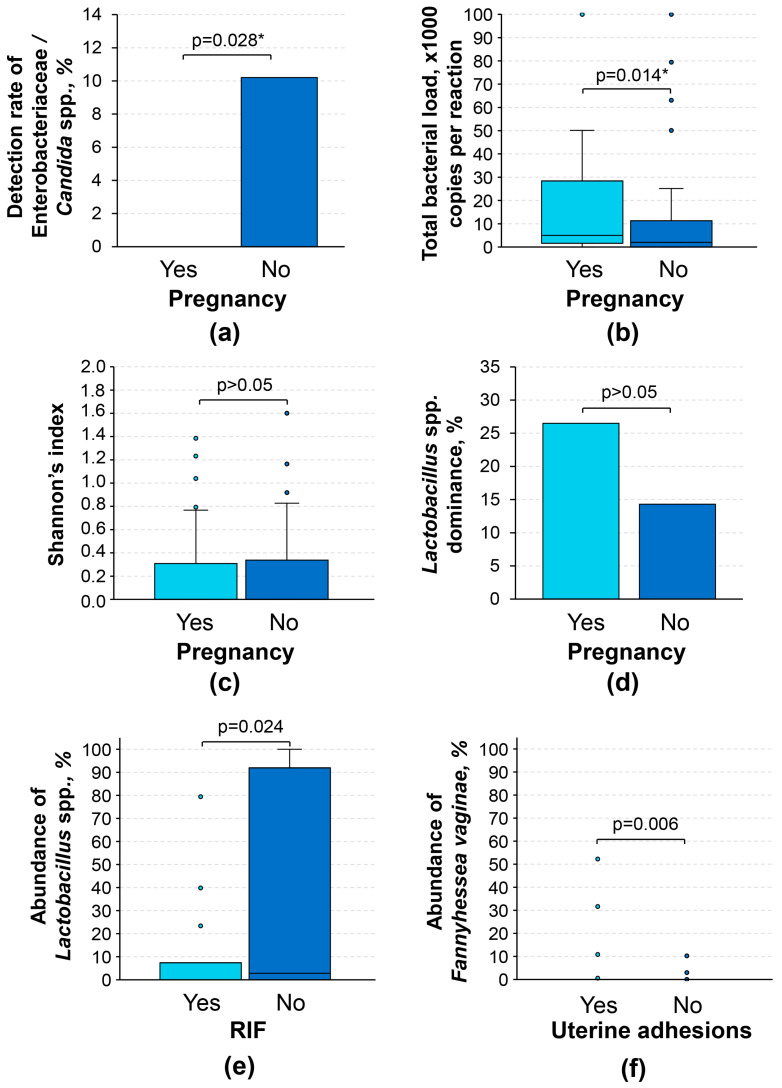
Associations between microbiological profiling results and clinical characteristics of study participants. (**a**) Detection rates of Enterobacteriaceae/*Candida* spp. based on the outcome of frozen ET; (**b**) boxplots for total bacterial DNA based on the outcome of frozen ET; (**c**) boxplots for Shannon’s indices based on the outcome of frozen ET; (**d**) detection rates of *Lactobacillus* spp. dominated communities based on the outcome of frozen ET; (**e**) boxplots for abundances of *Lactobacillus* spp. based on the presence of recurrent implantation failure; and (**f**) boxplots for abundances of *Fannyhessea vaginae* based on the presence of uterine adhesions. RIF, recurrent implantation failure. Bacterial abundances were calculated as % of the total bacterial load. “*Lactobacillus* spp. dominance” refers to a categorical variable that was positive when the abundance of *Lactobacillus* spp. was higher than the abundance of all other taxa combined. *p*-values for the comparisons of categorical variables were obtained using Fisher’s exact test, whereas for continuous variables, using Mann–Whitney’s U test. * This *p*-value did not pass the threshold for Benjamini–Hochberg correction for multiple comparisons at a false discovery rate of 0.2.

Investigation of several trends spotted during these comparisons revealed that menstrual blood samples with detectable *Candida* spp. and Enterobacteriaceae belonged exclusively to patients with negative outcomes of frozen ET (*p* = 0.028 *; Figure 2a), although these results must be interpreted as preliminary, as the sample size for positive cases was quite limited (*n* = 5). Interestingly, patients who achieved pregnancy after frozen ET had a slightly higher total bacterial load than the rest (1.6 × 10^3^ [5.0 × 10^3^; 2.8 × 10^4^] vs. 2.0 × 10^3^ [0.0; 1.1 × 10^4^] copies of bacterial genomes per reaction mixture; *p* = 0.014 *; Figure 2b).

Comparing the abundances of individual taxa was quite challenging; we evaluated two variables that are more suited for the interpretation of microbiological profiling studies, namely Shannon’s index and the *Lactobacillus* spp. dominance. The former is a quantitative variable that reflects α-diversity of the microbiological community, whereas the latter is a qualitative variable that is positive when the abundance of *Lactobacillus* spp. is higher than the abundance of all other taxa combined. Neither Shannon’s index nor the *Lactobacillus* spp. dominance was associated with the outcome of frozen ET (*p* > 0.05; Figure 2c,d). Notably, any attempts to account for the clinical and demographic features of the study participants listed in Table 1 using multivariable logistic regression did not alter the statistical significance of the results presented in this subsection. Therefore, the primary outcome of the study regarding the association of microbiological profiles of menstrual blood with post-frozen ET pregnancy rate was negative.

### 3.3. Association with Clinical Features

We investigated the associations of microbiological profiling results with various clinical features of the study participants, including common infertility factors. The abundance of *Lactobacillus* spp. was significantly lower in patients with recurrent implantation failure (RIF; defined as 3 or more implantation failures in anamnesis) than in patients without RIF (*p* = 0.024; Figure 2e). Patients with uterine adhesions had significantly higher abundances of *Fannyhessea vaginae* (*p* = 0.006; Figure 2f), although given the overall low detection rate of this microorganism (9.2%), these data must be interpreted with caution. We did not observe any other statistically significant associations between the clinical features listed in Table 1 and the results of microbiological profiling (*p* > 0.05).

## 4. Discussion

Microbiota is an essential component of the human organism and is involved in the regulation of many of its functions at both the local and systemic levels [16]. However, the intricacies of this interplay in the endometrium remain elusive due to various methodological and ethical challenges surrounding any research involving the assessment of ER. The approach based on the analysis of menstrual blood proposed in this study appears to be feasible in patients undergoing ART, as this biomaterial might be collected non-invasively at the beginning of the frozen ET cycle. This timing provides great opportunities to glimpse into the state of the endometrium within the target menstrual cycle’s boundaries (and to avoid dependency on results of previous cycles). Moreover, it potentially allows clinicians to obtain important data prior to embryo thawing (which is exceptionally useful in cases with limited availability of high-quality embryos) and to administer medications required for ET stimulation protocols [17]. According to the available literature, the proposed approach appears to be quite novel. Although menstrual blood has been actively researched and utilized as a source of mesenchymal stem cells for many years [18], it was largely overlooked for purposes of ER assessment. It is only recently that the first publications dedicated to the analysis of various signaling molecules in this biomaterial have appeared in the scientific literature [15,19,20].

In light of the latest research, the concept of a sterile endometrium has become actively disputed [13]. However, some researchers have suggested that microorganisms detected during the analysis of endometrial samples might be a result of contamination from the lower parts of the reproductive tract [21]. The previously mentioned problems of “splashome” and “kitome” might also be among the factors causing false-positive calls [14]. In our study, certain methodological steps were implemented to account for these phenomena, and it was proved that they did not have any impact on the microbiological profiling results presented here. There is an argument that the abundance of *Lactobacillus* spp. in the endometrium reported in a study might reflect the degree of contamination. A.D. Winters et al. performed 16S rRNA sequencing in endometrial samples (*n* = 25) obtained during hysterectomy and demonstrated that bacterial DNA was detectable in only 52% of cases, whereas *Lactobacillus* spp. was detectable in less than half of cases (median abundance of 0.006%), which is significantly lower than in most other studies where biomaterial was collected transcervically [22]. Similar results were obtained by C. Chen et al., who reported that *Lactobacillus* spp. have a limited abundance in endometrial samples collected at laparoscopy (median of 30.6%) [23]. Although menstrual blood was sampled through the cervix in our study, the selection of a conservative threshold for microorganism detection allowed us to avoid overdetection of this genus and bacterial DNA in general (detection rates of 46.9% and 75.5%, respectively, with *Lactobacillus* spp. abundance of 0 [0; 79.9] %), in concordance with the results above. Our previous report demonstrated that the endometrium significantly differs from the cervix and vagina in the detection rates of most taxa and Shannon’s α-diversity indices (lower in both cases) [24]. Therefore, it is expected that if menstrual blood had been contaminated by lower parts of the reproductive tract, the diversity of microbial communities reported in our samples would have been significantly higher. Moreover, we did not observe any statistically significant differences in detection rates and Shannon’s α-diversity indices in endometrial specimens obtained using biopsy and aspiration of menstrual blood (*p* > 0.05) in a large sample size (*n* = 198 for the combined cohort) [24], highlighting that aspiration of this biomaterial using an ET catheter is a promising alternative to traditional invasive sampling.

In recent years, the role of the endometrial microbiota in regulating receptivity has been increasing [13,25]. However, there is still no consensus on the exact characteristics of a microbial community that need to be interpreted to predict the outcomes of ART procedures. Some authors have proposed the abundance/detection rate of *Lactobacillus* spp. or α-diversity indices [26,27,28,29], whereas others report that these variables are not associated with pregnancy rates [30,31,32]. Interpretation and comparison of these results are hampered by pronounced methodological differences (inclusion criteria, IVF protocols, sampling, microbiological analysis techniques, data presentation, etc.) and small sample sizes in some cases; the former appears to be a barrier for meta-analyses as well. Results of microbiological profiling of menstrual blood were not associated with the outcome of frozen ET in our cohort. There was an interesting observation of the presence of Enterobacteriaceae and *Candida* spp., but given the limited sample size, these findings should be interpreted as preliminary and require further validation. Notably, the same trend regarding the detection of Enterobacteriaceae was reported by F. Cariati et al.; indeed, the dominance of these microorganisms in the endometrium can hardly be considered a healthy microbial community [32].

RIF, defined as an inability to achieve pregnancy after transferring three or more high-quality embryos, is among the main challenges of reproductive medicine because its etiology is often unknown, and treatment options are quite limited [33]. It is suggested that patients with RIF (and not the general population of patients in ART clinics) might receive the most benefit from microbiological assessment of the endometrium and antibiotic/probiotic treatment based on its results [34]. In our study, this condition was significantly associated with a reduced abundance of *Lactobacillus* spp., which is concordant with results of several other studies on the topic, although this observation requires further validation in larger, well-controlled studies [35,36]. Future interventional studies should probably focus on microbiological profiling with corresponding treatment in this particular subgroup of infertile patients.

There is evidence that intrauterine adhesions might cause shifts in the composition of endometrial microbiota, although not regarding *Fannyhessea vaginae*, as observed in our study [37,38]. This disease might lead to impaired ER among other conditions [39]. However, given the scarcity of data on the topic, the implications of these microbiota changes for reproductive health remain unclear and require further investigation.

This study has certain limitations. To begin, microbiological profiling was based on a multiplex assay for real-time PCR, which does not provide such a comprehensive characterization of microbial communities as sequencing-based approaches. Thus, our results should not be directly compared to findings of studies that utilized various sequencing techniques. The rationale for selecting multiplex real-time PCR instead of next-generation sequencing was based on two characteristics of the former. It is inexpensive and allows the analysis of the sample within a single day, whereas the turnaround time for microbiome analysis using sequencing is typically 1–3 weeks, which limits its potential to provide prognostically relevant data prior to embryo defrosting and hormonal stimulation [40,41,42]. Next, the sample size was limited due to the implementation of a novel methodology regarding the collection of endometrial biomaterials and the exploratory nature of this study, which rendered some statistical analyses underpowered (specifically for taxa with a detection rate below 10% at β = 0.20 and α = 0.05). Further validation of these results in an expanded cohort is required. Besides this, we did not apply any strict inclusion and exclusion criteria for the study enrollment; thus, the cohort was quite heterogeneous, and it was difficult to control all confounding factors due to the small sample size. Moreover, our methodology did not include cultivation of detected microorganisms, which limits the potential to draw conclusions regarding their role in ER regulation. Finally, this study focused on individuals undergoing frozen ET, and our findings should not be generalized for all IVF patients. It is known that frozen ET is associated with high rates of abnormal fetal growth and preeclampsia, whereas fresh blastocyst transfers are associated with low birthweight [43,44]. Therefore, exploration of endometrial microbiological profiles is a potential avenue for future research that might help uncover the pathophysiological basis of differences in fresh vs. frozen ET outcomes.

## 5. Conclusions

Menstrual blood aspirated directly from the uterus is a promising biomaterial for microbiological profiling of the endometrium. The non-invasive nature of its collection and the opportunity to obtain it within the investigated ET cycle may make this biomaterial feasible for ER studies involving ART clinic patients. Considering the results of the latest studies on the topic and the findings presented herein, the composition of microbial communities in the uterus might influence ER. However, given the lack of strong association of microbiological profiles and frozen ET outcomes, its role does not seem to be decisive and requires further validation in larger cohorts. Therefore, for future studies aiming to develop methods for predicting ART outcomes in infertile patients, it might be relevant to investigate endometrial microbiota as an addition to other ER biomarkers, such as cytokines, microRNA, and beyond.

## Figures and Tables

**Table 1:** Relevant demographic and clinical characteristics of the study participants.

Parameters	Pregnancy Group (*n* = 49)	No Pregnancy Group (*n* = 49)	*p*-Value
Age, years	34.8 (19.0–45.0)	38.0 (26.0–51.0)	0.040 *
Body mass index, kg/m^2^	22.5 (17.3–30.6)	22.4 (16.7–32.9)	>0.05
ART details: - IVF - ICSI - Stimulated cycle ^#^ - Natural cycle - PGT	55.1% 44.9% 59.2% 40.8% 53.1%	44.9% 55.1% 73.5% 26.5% 55.1%	>0.05 >0.05 >0.05 >0.05 >0.05
Infertility factors: - Tubo-peritoneal factor - Endometriosis - DOR - Uterine factor - Endocrinologic factor - Male factor - Genetic factor - Unknown factor	26.5% 10.2% 18.4% 4.1% 10.2% 26.5% 4.1% 10.2%	16.3% 28.6% 40.1% 8.2% 2.0% 20.4% 0.0% 14.3%	>0.05 0.039 * 0.026 * >0.05 >0.05 >0.05 >0.05 >0.05
Uterine adhesions	0%	2.0%	>0.05
Uterine fibroids	8.2%	10.2%	>0.05
RIF	18.4%	18.4%	>0.05
RPL	6.1%	4.1%	>0.05
Prolongation of pregnancy	93.6%	0.0%	N/A
Therapy in the last 3 months: - Antibiotics/antimicrobials - Vaginal pre-/pro- synbiotics	0.0% 0.0%	0.0% 0.0%	N/A N/A

Data are presented as the mean (range) or frequency (%). ART, assisted reproductive technologies; IVF, in vitro fertilization; ICSI, intracytoplasmic sperm injection; PGT, preimplantation genetic testing; DOR, diminished ovarian reserve; RIF, recurrent implantation failure; RPL, recurrent pregnancy loss; N/A, not applicable. RIF and RPL were defined as ≥3 corresponding events in anamnesis. Prolongation of pregnancy was claimed if pregnancy developed for at least 3 months. * This *p*-value did not pass the threshold for Benjamini–Hochberg correction for multiple comparisons at a false discovery rate of 0.2. ^#^ Hormonal regimens did not differ significantly between the groups (*p* > 0.05).

## Data Availability

All data generated in the present study are available in Table S1.

## Supplementary Materials

The following supporting information can be downloaded at: https://www.mdpi.com/article/10.3390/diagnostics16091403/s1, Table S1: Detailed results, clinical and demographic characteristics for all study participants.

## Abbreviations

The following abbreviations are used in this manuscript:

ART	Assisted reproductive technologies
ER	Endometrial receptivity
ET	Embryo transfer
FDR	False discovery rate
RPL	Recurrent pregnancy loss
RIF	Recurrent implantation failure
IVF	In vitro fertilization
PCR	Polymerase chain reaction
DOR	Diminished ovarian reserve
PGT	Preimplantation genetic testing
ICSI	Intracytoplasmic sperm injection
Spp.	Species
